# Projected Impacts of Climate and Land Use/Land Cover Change on Sediment Yield and Surface Runoff in the Baro River Sub‐Basin, Ethiopia

**DOI:** 10.1155/tswj/7891262

**Published:** 2026-06-24

**Authors:** Tewodros Getu Engida, Alemayehu Muluneh, Moltot Zewdie

**Affiliations:** ^1^ College of Engineering and Technology, Gambella University, Gambella, Ethiopia; ^2^ Faculty of Biosystems and Water Resources Engineering, Institute of Technology, Hawassa University, Hawassa, Ethiopia, hu.edu.et

**Keywords:** climate change, hydrological response, LULC change, sediment yield, SWAT

## Abstract

Understanding how climate change and land use/land cover (LULC) dynamics influence watershed hydrological processes is essential for achieving sustainable water and land management, particularly in data‐scarce and erosion‐prone basins. This study assesses the individual and synergistic effects of CMIP6‐based climate projections and LULC change on sediment yield and surface runoff in the Baro River sub‐basin. Utilizing a comprehensive hydrological modeling framework, this study integrates the Soil and Water Assessment Tool (SWAT) with CMIP6 multi‐model ensemble climate projections under SSP2‐4.5 and SSP5‐8.5 scenarios to simulate runoff in addition to sediment yield. We projected future LULC dynamics (2024–2064) using a multi‐layer perceptron–cellular automata–Markov chain (MLP‐CA‐MC) model. Results indicate statistically significant warming in both historical and future periods, with mean temperature increases of up to 3.4°C under SSP5‐8.5, alongside precipitation increases of 6%–13%. LULC projections (2024–2064) indicate an 18%–26% increase in agricultural and settlement areas at the expense of forest, grassland, and shrubland. The SWAT model demonstrated high predictive accuracy; Nash–Sutcliffe efficiency (NSE) values remained strong across both calibration (runoff: > 0.87; sediment: > 0.76) and validation (runoff: 0.84; sediment: 0.73). Projected sediment yield increases from a 2024 baseline of 49.23 t/ha to as high as 157.68 t/ha over the late period (2072–2100) under the SSP5‐8.5 scenario with 2064 land use conditions. Similarly, surface runoff is expected to peak at 966.58 mm under high‐emission conditions. These results highlight the critical sensitivity of the Baro River sub‐basin to both climate variability and anthropogenic land degradation, underscoring the urgent need for integrated watershed management to mitigate downstream sedimentation and ensure the sustainability of water infrastructure.

## 1. Introduction

In the last few decades, river basins worldwide have been undergoing sharp changes in their hydrological behavior attributed to the joint impact of climate change and rapid land use/land cover (LULC) changes [1]. Climate change and LULC change are the two most significant factors impacting the hydrological cycle, which lead to shifts in the amount, timing, and distribution of rainfall, information essential for water resource planning and utilization [2, 3]. In East Africa, these changes threaten ecological sustainability and agricultural productivity, particularly in Ethiopia [4]. For this reason, one has to account for all the changes that could occur with a changing climate and rapid LULC variation.

Global warming has emerged as one of the principal environmental problems [5, 6], strongly increasing the intensity of extreme rainfall events and hydrological processes globally [7].

The speed of increase in temperatures around the globe has become exceedingly fast in recent years. Although the overall global temperature on average has been increasing by 0.08°C per decade since 1880, its increase from 1981 onward has more than doubled. Over the last four decades, the general warming trend has been 0.18°C per decade [8]. IPCC [9] reported that the increase in global surface temperature is destabilizing the climate system and intensifying the hydrological cycle. The shift has led to an increase in the frequency and severity of extreme hydrological events such as floods and droughts. Several scientific researchers also show that an increase in overall surface temperature has led to alterations in the global hydrological cycle, leading to significant shifts in the temporal and spatial distribution of water resources and precipitation [10–12]. In a local watershed, the stream flow alters nonlinearly with changes in precipitation events [13].

Based on the study by Wang et al. [14], in dry regions, a rise in surface temperature is linked to an increase in evaporation and transpiration, which can explain variations in runoff and stream flow. However, in moist areas, a change in precipitation causes variation in stream flow and sediment. The nonlinear relationship between stream flow and sediment load (SL) means that precipitation extremes and stream flow apply a disproportionate influence on total sediment transport [15]. In many catchments, short‐duration and intense precipitation events are principally responsible for a large portion of the total sediment transport [15, 16]. While the effects of changing precipitation on soil erosion are complex and are not invariably negative, increasing rainfall can drive soil erosion, but it also helps vegetation growth and canopy cover, which naturally protect the soil and reduce runoff ([17]).

Simultaneously, rapid industrial and socioeconomic advancements have intensified human‐driven LULC changes, precipitating major ecological challenges [18]. LULC changes affected by human activities might redistribute hydrological patterns by altering percolation, infiltration, evaporation, and groundwater flow. These shifts significantly influence sediment transport and watershed hydrology [19, 20].

Due to recent geopolitical and socioeconomic changes, the profound impact of climate change and LULC on local watershed hydrology has become a global research priority. Several studies have been carried out on the separate or synergistic effects of climate or LULC change on stream flow [21–25]; similarly, studies on sediment yield (SY) have been carried out [13, 26, 27]. Similar studies have been conducted in Ethiopia in various watersheds [28–34].

In sub‐Saharan Africa, and Ethiopia in particular, watersheds are extremely susceptible due to strong dependence on rain‐fed farming, rapid population growth, and ongoing land transformation. The Baro River sub‐basin, a major tributary of the Sobat–White Nile system, plays a crucial role in regional water availability, hydropower development, irrigation expansion, and ecosystem services. However, the basin has experienced substantial deforestation, agricultural expansion, and settlement growth over recent decades, which, combined with climate variability, have intensified runoff generation and SY. These pressures threaten the sustainability of existing and planned water infrastructure and increase downstream sedimentation risks.

Extensive research has been conducted in the Baro–Akobo River basin to assess the individual impacts of climate and LULC dynamics on local hydrology [35–37]. While certain scholars have explored synergistic co‐effects of these factors on the water balance, they have largely relied on CMIP5 general circulation models (GCMs) [37]. These older models often suffer from coarse resolution, and previous projections have shown significant uncertainty regarding the magnitude and direction of mean rainfall across various regions [36]. This lack of precision hinders the reliability of hydrological projections in a region where rainfall variability is high.

There is a pressing need to enhance GCM selection and hydrological projections for the Baro River sub‐basin by adopting the latest Coupled Model Intercomparison Project Phase 6 (CMIP6) models under shared socioeconomic pathway (SSP) scenarios. Currently, no studies have utilized CMIP6 to investigate projected impacts specifically on SY and water balance in this region. This is a critical omission, as the lower Baro River, particularly in the Lare, Wantuwa, Itang, Abol, and NibNib woredas, as well as Gambella Town, has been severely impacted by sedimentation, riverbank expansion, and flood risks over the last decade.

Furthermore, few studies integrate historical and future LULC changes with the latest CMIP6 climate projections. Therefore, comprehensive scientific research is required to investigate the combined and separate effects of climate and LULC change on SL in addition to flood hazards in the Baro River sub‐basin using a CMIP6 climate model, multi‐layer perceptron–cellular automata–Markov chain (MLP‐CA‐MC), and a hydrological model.

Therefore, the objectives of this research are as follows: (1) to analyze historical and future climate trends using CMIP6 multi‐model ensemble (MME) projections under SSP2‐4.5 and SSP5‐8.5 scenarios, (2) to evaluate historical and future LULC dynamics using the MLP‐CA‐MC model, and (3) to assess the individual and combined impacts of climate change and LULC change on SY and stream flow.

The findings of this study provide a comprehensive understanding of how sediment responds to variations in climate and LULC change, and this knowledge is critical in developing strategic techniques for managing hydrology and resource allocation in the Baro River sub‐basin.

## 2. Material and Methods

### 2.1. Description of the Study Site

**Figure 1:**
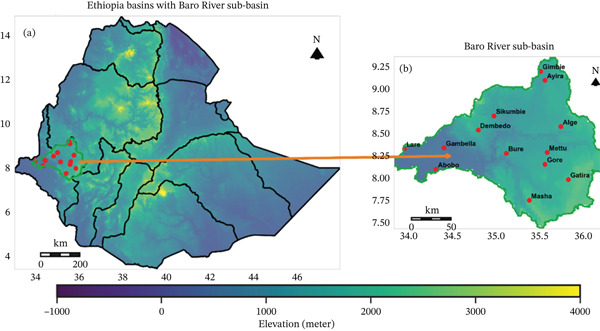
Location map of the study site with the digital elevation model (DEM) in meters.

The site is situated within the Baro–Akobo River basin in southwestern Ethiopia; the Baro River sub‐basin spans from 7.4550° to 9.3767° N latitude and from 33.8° to 36.4° E longitude. The study site covers approximately 26,756 km^2^ of drainage area. The Baro River sub‐basin tributaries flow into the Baro–Akobo River basin in the direction of South Sudan. The stream flow outlet is located at the geographic coordinates of 8.2° N latitude and 32.82° E longitude below the Baro Gambella River Bridge. Surface elevation ranges from 366 to 3266 m above sea level, showing substantial variation in the topography of the study area (Figure 1).

The Baro River sub‐basin is characterized by a tropical monsoon climate that is well‐defined by high spatial and seasonal variability. The wet season commonly extends from March to October, whereas the dry season extends from November to February; however, the timing differs by surface elevation: the uplands receive precipitation from mid‐April to October, whereas the lowlands receive precipitation mainly from mid‐June to September and in March. Yearly precipitation averages 1500 mm, ranging from 1100 to 1900 mm. Surface temperature extremes are most pronounced between March and April; spatially, average annual minimums rise from 15°C in the western highlands to 23°C in the eastern lowlands, with maximums following a similar gradient from 26°C to 36°C.

The elevation of the Baro River sub‐basin ranges from 366 to 3266 m above mean sea level. The Baro River sub‐basin ranges from high‐cold mountainous areas to semi‐desert lowland areas, with extreme ranges of temperature and precipitation. The Baro River sub‐basin is characterized by a long rainy season based on the movement of the intertropical convergence zone (ITCZ). The wet season can be considered monomodal, bimodal, or trimodal in rainfall pattern, with a well‐defined rainy season from mid‐April to October and a dry season from November to February.

### 2.2. Method of Analyzing Historical and Projected Climate Trends in Temperature and Precipitation

#### 2.2.1. Observed Climate Data and Analysis

The historical observed climate data for the study site, such as precipitation, temperature, humidity, wind speed, and sunshine duration data from the period 1986–2024, were provided by the Ethiopian Meteorological Institute. Data collection was done from 13 stations within the Baro River sub‐basin, as shown in Figure 1.

In order to ensure spatial representativeness for the Baro River basin, which comprises different topographies ranging from highlands to lowlands, climate data were systematically collected from 13 stations located within the basin and its immediate surroundings. The historical period was chosen for the climate data, ranging from 1986 to 2014, to enable precise bias correction by aligning with the CMIP6 historical baseline. Double mass analysis was used to ensure the uniformity of the precipitation data, and missing data were estimated using the arithmetic mean and normal ratio. Furthermore, the Thiessen polygon method was used to ensure the uniform distribution of the climate data.

#### 2.2.2. Future Climate Data

Daily precipitation and temperature data, including daily minimum and maximum values, were obtained from CMIP6 via the Copernicus Climate Change Service.

The selection of GCMs was based on data completeness, specifically identifying models that provided all required elements for both the SSP2‐4.5 and SSP5‐8.5 scenarios within the study region. Consequently, a total of 12 GCMs were utilized for this analysis, as detailed in Table 1.

**Table 1:** CMIP6 models used in this study with their spatial resolutions and sources.

CMIP6 model	Resolution (°)	Key reference
ACCESS‐CM2	1.875^°^ × 1.25^°^	[38]
BCC‐CSM2‐MR	1.125^°^ × 1.125^°^	[39]
CanESM5	2.8^°^ × 2.8^°^	[40]
EC‐Earth3‐Veg‐LR	1.125^°^ × 1.125^°^	[41]
FGOALS‐g3	1.0^°^ × 1.0^°^	[42]
GFDL‐ESM4	1.25^°^ × 1.0^°^	[43]
INM‐CM5‐0	2.0^°^ × 1.5^°^	[44]
IPSL‐CM6A‐LR	2.5^°^ × 1.25^°^	[45]
MIROC6	1.4^°^ × 1.4^°^	[46]
MIROC‐ES2L	2.812^°^ × 2.812^°^	[47]
MPI‐ESM1‐2‐LR	1.875^°^ × 1.875^°^	[48]
NorESM2‐MM	1.25^°^ × 0.9375^°^	[49]

The CMIP6 model projections for the study site were obtained in the form of NetCDF files, which were then passed through a rigorous pre‐processing scheme to ensure consistency among the models. Using the Climate Data Operators (CDO) tool, the global datasets were clipped spatially to the specific boundaries of the study area. To account for the varying resolution of the individual models, bilinear interpolation was used to ensure that all the datasets were interpolated within a standard grid resolution of 0.5^°^ × 0.5^°^. To reduce the uncertainties of individual models and obtain a balanced perspective of the future climate paths, the MME approach was employed. The MME combined 12 different GCMs, with one model realization used per model, along with an equal weighting approach.

##### 2.2.2.1. Bias Correction of Climate Model Data

While GCMs are very vital in predicting climate behavior at a global scale, they tend to have some systematic errors in terms of their ability to reproduce local precipitation and temperature patterns. In order to get accurate and region‐specific climate predictions, the biases need to be corrected. One such method that has been adopted is called quantile mapping (QM). We utilize QM, which functions as a form of statistical downscaling. The QM transformation entails setting the cumulative distribution function (CDF) of the target variable to be equal to the CDF of the observational equivalent. The transformation can be mathematically described as follows:

x0=fxm,

where *x*_0_ represents the bias‐corrected (observed‐equivalent) variable, *x*_m_ is the raw modeled variable, and *f*( ) is the transformation function, which is derived from the statistical relationship between observed and modeled distributions. More specifically, QM applies the inverse CDF (quantile function) of the observed dataset with the CDF of the modeled data, written as follows:

x0=F0−1Fmxm.

Here, *F*_m_(*x*_m_) is the CDF of the modeled variable, and $F_{0}^{-1}$ denotes the inverse CDF (quantile function) of the observed data. This technique effectively adjusts the distribution of the model output to more closely match observed climatology, thereby enhancing the reliability of the simulations. Numerous studies have demonstrated the effectiveness of QM [50–52], particularly nonparametric methods, in reducing biases in regional climate model (RCM) outputs, especially for precipitation. The bias adjustment process involved the use of QM, which is a distributional approach that matches the CDFs between the CMIP6 historical simulation dataset and the observations based on the reference period (e.g., 1981–2014). The main features of the process include the following: (i) the use of 100 quantiles for capturing the tail behavior of distributions required for extremes and (ii) monthly adjustment for seasonality in model bias. In this study, a total of 12 climate models containing monthly‐scale precipitation were selected based on two future SSPs (SSP2‐4.5 and SSP5‐8.5). The data from each model were combined onto a 0.5^°^ × 0.5^°^ grid using a bilinear interpolation system. The deviation of the model data was corrected by the observed data based on the equiratio CDF matching method, and the baseline period of calibration was 1986–2014. The 12 bias‐corrected climate datasets were finally pooled into one model (MME_correct) using the MME arithmetic mean method.

##### 2.2.2.2. Climate Trend Analysis

For rainfall trend analysis, the Mann–Kendall test statistic was used. The Mann–Kendall test is a nonparametric rank‐based test for determining the nature of a trend in a particular time series with respect to the null hypothesis that no trend is present [53, 54]. The use of the test is becoming increasingly popular for analyzing hydrological data from East Africa [55, 56].

An analysis of rainfall and temperature change over a multi‐decadal period will be carried out to identify climate change anomalies and interannual and interseasonal changes with respect to the historical period. The rainfall and temperature for the future period will be divided into three time slices: 2021–2040, 2041–2060, and 2081–2100. For climate change, changes in rainfall and temperature for the coming period (2021–2100) with respect to the SSP2‐4.5 and SSP5‐8.5 scenarios and the baseline period (1986–2014) will be used.

### 2.3. Method of Analysis for Historical and Future LULC

#### 2.3.1. Analysis of Historical LULC Detection

The multi‐temporal imagery was downloaded from the United States Geological Survey (USGS) EarthExplorer website (https://earthexplorer.usgs.gov). The multi‐temporal imagery used archived datasets; specifically, Landsat 8 imagery was used to collect a reliable dataset for the 2004, 2014, and 2024 study periods, as shown in Table 2.

**Table 2:** Data type and source for historical LULC detection.

Data type	Description	Data source
Satellite imagery	Landsat 8 (OLI)	USGS EarthExplorer (https://earthexplorer.usgs.gov)
Reference data	Ground truth points (GTP) for accuracy assessment	Field surveys, Google Earth Pro

The research design adopted a unique integration of primary and secondary satellite imagery for the classification process, ensuring the accuracy of the results for the investigation of past and present LULC.

##### 2.3.1.1. LULC Classification and Accuracy Assessment

The pre‐processing workflow was implemented using raw satellite imagery in ERDAS Imagine 16 software. This was done following the existing guidelines by Islam et al. (2018) on the protocols to be followed. The imagery was subjected to radiometric correction and geometric correction to ensure spectral integrity. A supervised digital image classification was done using the maximum likelihood classifier (MLC) algorithm, categorizing the study area into seven distinct classes: water body, grassland, forest land, farmland, bare land, settlement, and shrubland. To ensure that scientific standards were met for the 2024 maps, the classification was integrated with the following: field surveys, which provided information on the types of land use, and Google Earth observations, which provided additional information on the differentiation of the clusters. Finally, an accuracy assessment was conducted. Overall accuracy (OA), user accuracy (UA), producer accuracy (PA), and kappa coefficient (Kappa) were calculated using the mathematical equations below and applying the stratified sampling technique. This was done to ensure that the maps reached the scientific standard of at least 85%, as suggested by Foody [57].

→ Overall accuracy (OA):

OA=∑i=1rxN,

→ Producer accuracy (PA):

PA=xiix+i,

→ User accuracy (UA):

UA=xiix+,

→ Kappa coefficient (Kappa):

Kappa=N∑i=1rxii−∑i=1rxi+i∗x+1N2−∑i=1rxi+∗x+i,

where

→ *N* is the total number of pixels or samples.
→ *r* is the number of rows/columns in the matrix.
→ *x*_*i* *i*_ represents the diagonal elements (correctly classified samples).
→ *x*_*i*+_ is the sum of row *i* (total predicted for a class).
→ *x*_+*i*_ is the sum of column *i* (total ground truth for a class).

#### 2.3.2. Future LULC Projection

##### 2.3.2.1. Driving Factors

For the simulation of transition probabilities of future land use, the process of data acquisition incorporated a set of necessary socioeconomic and environmental variables. The set of explanatory variables, which were classified into topographic variables, proximity variables, and demographic variables, was used in the MLP approach to detect spatial variations of change. The datasets and their sources are described in Table 3.

**Table 3:** Data type and source for projected future LULC detection.

Data category	Data type	Description	Data source
Topographic	DEM	Digital elevation model (used to derive slope and aspect)	USGS EarthExplorer (https://earthexplorer.usgs.gov)
Spatial	Proximity (distance factors)	Distance to roads, distance to existing settlements, and distance to water bodies	EthioGIS
Demographic	Population	Population density often correlates with built‐up expansion	Ethiopian Central Statistical Agency

##### 2.3.2.2. Model Framework (MLP‐CA‐MC)

To simulate the future landscape pattern for the years 2034, 2044, and 2064, the integrated modeling approach was used, which combines MLP, CA, and MC. TerrSet software Version 18.31 was used to apply the land change modeler (LCM) to simulate future LULC changes based on past trends between 2004 and 2014. The stepwise approach by Eastman (2016a) was followed by integrating the driver variables into the LCM’s sub‐model for transition. An MLP‐NN was used to generate the transition potential maps. The maps successfully simulated the suitability of land transformation by incorporating the effects of the selected driver variables [58–60]. Next, a hybrid CA‐MC model was applied to simulate the future LULC changes up to the year 2064.

To ensure the accuracy of the model’s prediction, the output for the year 2024 was validated with a reference map for the actual year 2024 and then calibrated for future scenario projections. Once calibrated, this validated model was applied to project spatial LULC distributions for the long‐term horizons of 2034, 2044, and 2064.

##### 2.3.2.3. Performance Assessment of the MLP‐CA‐MC Simulation

This validation process was done to check the current level of agreement and disagreement between the real satellite‐driven (Mp1) and simulated (Mp1 ^′^) LULC maps of 2024, for the purpose of proving the accuracy of this MLP‐CA‐MC model in predicting the future maps of 2034, 2044, and 2064 [61]. For this experiment, the VALIDATE module was used with hard and soft predictions. The VALIDATE module was used in this case for performing the validation process. The VALIDATE module calculates kappa statistics using the Mp1 ^′^hard prediction as a comparison map: Kappa for no information (*K*_no_), Kappa for grid‐cell‐level location (*K*_location_), Kappa for stratum‐level location (*K*_locationStrata_), and Kappa standard (*K*_standard_) [62, 63]. An appropriate value of AUC and Kappa is considered to be close to 80%. In future prediction, the 2004 and 2014 images were chosen as the independent variables to predict the LULC maps of 2034, 2044, and 2064.

#### 2.3.3. Rate of Change Analysis

To compute the magnitude of these transitions, the annual rate of change (*r*) is determined. This study utilizes the standard compound growth equation, a methodology frequently endorsed by the Food and Agriculture Organization (FAO). To understand the intensity of these changes, we calculate the annual rate of change (*r*) using the standard compound interest formula often used by the FAO:

r=A2A11/year−1∗100,

where *A*_1_ and *A*_2_ are the land use percentage at the start and end of the period, and *t* is the number of years.

### 2.4. Method of Data Analysis for Sediment Modeling

#### 2.4.1. Soil and Water Assessment Tool (SWAT) Model Description

The SWAT is a physically based, semi‐distributed, and process‐oriented model that was developed by the United States Department of Agriculture (USDA). The SWAT model operates on a continuous and daily time scale. The SWAT model was specifically engineered to perform catchment‐scale simulations to investigate the long‐term effects of land management practices on surface runoff (SR), base flow, SY, and water quality in large and complex catchments [64, 65].

The ArcSWAT interface was employed to simulate the Baro River sub‐basin. The modified soil conservation service (SCS) curve number (CN) method was employed to compute the runoff amounts based on precipitation data. The execution of the SWAT model depends on the essential spatial and climatic datasets such as the DEM, LULC, and meteorological data.

#### 2.4.2. Data Type and Source for the SWAT Model

Modeling the Baro River sub‐basin’s SY and SR process via SWAT requires a variety of technical datasets. These inputs were divided into three categories: spatial, meteorological, and hydrological. The data types used for model application are described in Table 4.

**Table 4:** Data type and source of SWAT model simulation.

Data category	Data type	Description	Data source
Spatial	DEM	SRTM (30 m∗30 m)	USGS EarthExplorer (https://earthexplorer.usgs.gov)
Spatial	LULC	Landsat 8 (OLI)	USGS EarthExplorer
Spatial	Soil data	Shapefile (physical/chemical properties)	Ethiopian Ministry of Water and Energy (MoWE)
Meteorological	Climate (historical and projected)	Daily precipitation, Tmax/Tmin, solar radiation, and wind speed	National Meteorological Agency (NMA) of Ethiopia
			Copernicus climate data
Hydrological	Calibration and validation data	Daily river discharge and sediment	Ethiopian Ministry of Water and Energy (MoWE)

#### 2.4.3. ArcSWAT Setup

##### 2.4.3.1. Watershed Delineation

Watershed delineation features in ArcSWAT 2012 enable the user to delineate the watershed and sub‐watersheds through the use of the DEM of the study site. This particular feature relies on the expanded ArcGIS 10.3 spatial analysis extension. The flow direction and accumulation determine how the stream network is defined from the DEM within the SWAT framework. The source of the stream depends on its threshold area, which is used to generate the source of streams and ultimately define the sub‐basin or sub‐watershed. The monitoring point was added manually, and the number of sub‐basins was adjusted accordingly, resulting in the establishment of 37 sub‐catchments.

##### 2.4.3.2. Hydrologic Response Unit (HRU) Analysis

After watershed delineation, the spatial variability of the watershed was subsequently defined further based on the definition of the HRUs. The HRUs are homogeneous portions of the land that have the same combination of land use type, soil, and slope.

In order to maintain consistency with the SWAT database, soil, LULC, slope, and topographic datasets were processed through an extensive data integration and reclassification procedure.

###### 2.4.3.2.1. Soil Data

The soil dataset was first converted to a shapefile and related to the user soil database using a secondary lookup table.

Spatial data of soil form one of the basic elements of the SWAT model, which requires extensive physicochemical data such as texture, bulk density, hydraulic conductivity, water holding capacity, and organic carbon, among others, for all layers. For linking this data to the Baro River sub‐basin, a unique lookup table had to be developed, which is basically a relational tool for making an association between spatial data of soils in GIS and the name of soils (SNAM) in the SWAT user database. Map values, soil classes, and SWAT codes are correlated in Table 5.

**Table 5:** Soil SWAT codes.

Value	Soil name (SNAM)	Value	Soil name (SNAM)
1	Dystric Nitisols	10	Leptosols
2	Dystric Gleysols	11	Eutric Cambisols
3	Chromic Vertisols	12	Dystric Cambisols
4	Orthic Acrisols	13	Gypsic Yermosols
5	Eutric Nitisols	14	Dystric Fluvisols
6	Chromic Luvisols	15	Cambisols
7	Eutric Fluvisols	16	Calcaric Fluvisols
8	Orthic Solonchaks	17	Calcic Cambisols
9	Calcic Fluvisols	18	Calcic Xerosols

###### 2.4.3.2.2. LULC Integration

In order to include the data on LULC in the SWAT model for the Baro River sub‐basin, reclassification of data was done using a special lookup table. This is necessary for the precise calculation of SR and other processes occurring within each individual HRU. Map values, LULC classes, and SWAT codes are correlated in Table 6.

**Table 6:** LULC class and SWAT code.

Value	LULC class	SWAT database	SWAT code
1	Forest land	Forest‐Evergreen	FRSE
2	Shrubland	Range‐Brush	RNGB
3	Agricultural land	Agricultural Land‐Generic	AGRL
4	Grassland	Range‐Grasses	RNGE
5	Water body	Water	WATR
6	Settlement	Residential‐Medium Density	URMD
7	Bare land	Barren	BARR

A lookup table was created to establish the correspondence between land uses in the area and SWAT codes consisting of four letters.

###### 2.4.3.2.3. Slope Classification

Based on Table 7, a multiple‐slope approach was adopted (an option for considering different slope classes for HRU definition), and the terrain was classified into five slope classes.

**Table 7:** Slope classes in the Baro River sub‐basin.

Slope classes (%)	Area (coverage) (km^2^)	Watershed area (%)
0–5	4335.327	17.6397
5–10	5917.326	24.07659
10–15	5793.173	23.571
15–20	3670.982	14.9366
20–9999	4860.288	19.77568

###### 2.4.3.2.4. HRU Definition and Thresholding

Once the reclassification of the LULC, soil, and slope maps had been overlaid, the thresholding technique was used to enhance computational efficiency by removing unnecessary landscape features that have little effect on the hydrological water budget. For the Upper Baro basin, a choice of multiple HRUs was made. In order to achieve high resolution, the thresholds for LULC, soil, and slope were selected as 5%, 5%, and 10%, respectively.

##### 2.4.3.3. Meteorological Inputs

Daily observed climate data, including precipitation, maximum/minimum temperatures, humidity, wind speed, and sunshine hours, were obtained from the Ethiopian Meteorological Institute for 13 stations within the Baro River sub‐basin. The SWAT weather generator model (WXGEN) was used to fill missing weather data values. Precipitation and temperatures at all stations were prepared in comma‐delimited (.csv) format over the Baro River sub‐basin. However, solar radiation, relative humidity, and wind speed data were available only for the principal Gore and Masha stations. The daily meteorological dataset (precipitation and minimum/maximum air temperatures on a daily basis), with the missing data replaced by the missing value indicator −99, and the location tables prepared based on the SWAT format were loaded into the model.

#### 2.4.4. Hydrological Component of SWAT

The hydrological cycle simulated by SWAT is based on the water balance equation [66].

SWt=SW0+∑i=1tRday−Qsurf−Ea−Wseep−Qgw,

where

→ SW_*t*_ = final soil water content (mm)
→ SW_0_ = initial soil water content on day *i* (mm)
→ *t* = time (day)
→ *R*_day_ = amount of precipitation on day *i* (mm)
→ *Q*_surf_ = amount of SR on day *i* (mm)
→ *E*_a_ = amount of evaporation on day *i* (mm)
→ *W*_seep_ = amount of water entering the vadose zone from the profile on day *i* (mm)
→ *Q*_gw_ = amount of return flow on day *i* (mm)

##### 2.4.4.1. SR Generation

SWAT 2005 provides two SR computation methods: a modification of the SCS CN method (USDA SCS, 1972) or the Green and Ampt infiltration method [67, 68], as cited in Ndomba et al. [69].

Qsurf=Rday−0.2S2Rday−0.8S,

where *Q*_surf_ is the accumulated daily SR (mm), *R*_day_ is the daily rainfall (mm), and *S* is a retention parameter.

The parameter *S* is related to CN and was estimated by the following equation:

S=25.4∗100CN−10,

where CN is the curve number for day *i*, and its value is a function of land use practice, soil permeability, and soil hydrologic group.

### 2.5. Sediment Estimation

Datasets on hydrology and sediments used in the study were sourced from the Ministry of Water, Irrigation, and Energy (MoWIE). Stream flow data spanning 31 years (1990–2020) and sediment data at different times were obtained at the Baro Gambella station.

#### 2.5.1. Sediment Rating Curve (SRC)

In the absence of continuous suspended sediment records for the Baro River sub‐basin, an SRC was established to estimate missing values. This technique defines the empirical relationship between river discharge and sediment concentration or load, serving as a standard method for approximating sediment transport. The curve was developed by correlating measured sediment data with concurrent stream flow observations at the Baro Gambella gauging station [64]. The SRC is a widely used technique for estimating suspended SL by defining the relationship between river discharge and sediment concentration or load [64].

The relationship is typically represented using a mathematical curve‐fitting power function:

SL=a∗Qb,

where

→ SL = sediment load (ton/day)
→ *Q* = stream discharge (m3/s)
→ *a* and *b* = regression constants determined through data analysis

To convert the measured suspended sediment concentration (*C*) in mg/L from the Baro Gambella station into daily SL in ton/day, the following conversion equation was utilized:

SL=0.0864∗Q∗C,

where

→ SL = sediment load (ton/day)
→ *Q* = discharge (m3/s)
→ *C* = suspended sediment concentration (mg/L)
→ 0.0864 = factor used for unit conversion

#### 2.5.2. SY Estimation

The SWAT model uses the modified universal soil loss equation (MUSLE) to predict the amount of SY for a given HRU [70]. This approach is much more accurate because the rainfall energy factor is replaced with a runoff factor in the USLE. This is because the runoff factor takes into account the antecedent moisture and rainfall energies.

SED=11.8Qsurf∗Qpeak∗areaHRU0.56∗KUSLE∗CUSLE∗PUSLE∗LSUSLE∗CFRG,

where

→ SED is the SY on a given day (metric tons).
→ *Q*_surf_ is the SR volume (mm).
→ *Q*_peak_ is the peak runoff rate (m3/s).
→ area_HRU_ is the area of the HRU (ha).
→ *K*_USLE_ is the USLE soil erodibility factor (0.013 metric ton m2 h/m3 metric ton cm).
→ *C*_USLE_ is the USLE cover and management factor.
→ *P*_USLE_ is the USLE support practice factor.
→ LS_USLE_ is the USLE topographic factor.
→ CFRG is the coarse fragment factor.

### 2.6. Conceptual Framework for Sediment Estimation

**Figure 2:**
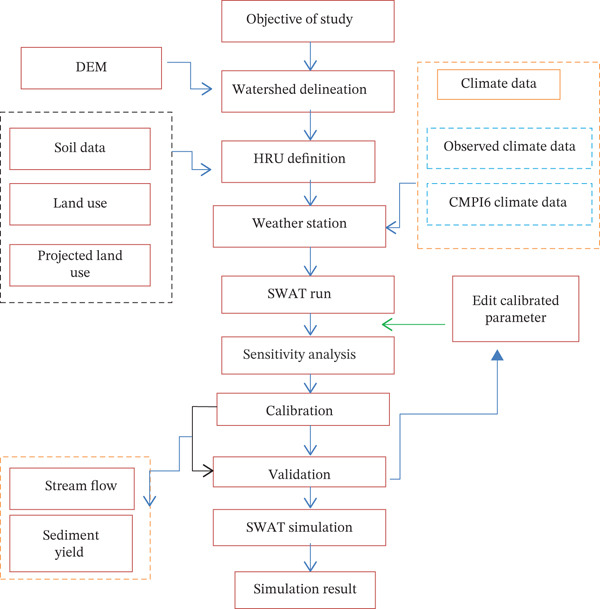
Methodological framework for modeling sediment yield in the Baro River sub‐basin.

The methodological approach for this research is structured through a systematic conceptual framework, which outlines the integration of spatial and hydro‐climatic data within the SWAT model environment. As illustrated in Figure 2, the workflow proceeds from initial data acquisition and watershed characterization to model execution, performance evaluation, and final result interpretation.

### 2.7. Model Calibration and Validation

The hydrological data used for the current research were acquired from the MoWIE. The data acquired consist of daily stream flow data and sediment data obtained through rating curves. These data have been used over a 31‐year period, spanning from January 1, 1990, to December 31, 2020. These data were acquired at the Baro Gambella gauging station.

### 2.8. Model Performance Evaluation

We employed the Moriasi et al. [71] framework to validate the model’s predictive accuracy. This involved calculating the Nash–Sutcliffe efficiency (NSE), percent bias (PBIAS), and the coefficient of determination (*R* ^2^) to ensure the simulation of hydrological dynamics was statistically sound.

Based on the Moriasi et al. [71] guidelines, model performance is categorized into four levels of adequacy:

➢ 0.75 < *N* *S* *E* < 1.00, *R*2 > 0.85, *P* *B* *I* *A* *S* < ±10—very good
➢ 0.65 < *N* *S* *E* ≤ 0.75, 0.75 < *R*2 ≤ 0.85, ±10 < *P* *B* *I* *A* *S* < ±15—good
➢ 0.50 < *N* *S* *E* ≤ 0.65, 0.60 < *R*2 ≤ 0.75, ±15 < *P* *B* *I* *A* *S* < ±25—satisfactory
➢ *N* *S* *E* ≤ 0.50, *R*2 < 0.60, *P* *B* *I* *A* *S* > ±25—unsatisfactory

### 2.9. Separate and Combined Impact Assessment for Hydrological Processes and SY

#### 2.9.1. Impact Assessment for Hydrology and Sediment

To assess the factors that influence hydrological change in the Baro River sub‐basin, this study examines the separate and synergistic effects of climate change and LULC change on stream flow and SY. The assessment is conducted for three future periods: near‐term (2015–2043), mid‐term (2044–2071), and long‐term (2072–2100) periods, as well as for the SSP2‐4.5 and SSP5‐8.5 climate scenarios. The “one‐factor‐at‐a‐time” method was used to determine the individual effects of the factors. By fixing one factor (either climate or LULC) and comparing it with the other, which is varied relative to the 1986–2014 baseline, the relative effect of each factor can be determined. Major categories of simulations were developed to enable this comparison.

Table 8 provides the simulation scenarios that are required to identify the driving factors in the Baro River sub‐basin.

**Table 8:** Framework for LULC and climate change impact analysis.

Impact analysis	Simulation input configuration
Climate change effect on hydrology	Projected climate for Periods A, B, and C combined with static 2024 baseline LULC data under SSP2‐4.5 and SSP5‐8.5.
Climate change effect on sediment yield	Projected climate for Periods A, B, and C combined with static 2024 baseline LULC data under SSP2‐4.5 and SSP5‐8.5.
LULC change effect on hydrology	Projected LULC for 2024, 2034, 2044, and 2064 combined with static baseline climate data (1986–2014).
LULC change effect on sediment yield	Projected LULC for 2024, 2034, 2044, and 2064 combined with static baseline climate data (1986–2014).
Combined effects of climate change and LULC on hydrology	Integrated simulation using both projected climate (A, B, and C) and projected LULC (2024–2064) across both SSP scenarios.
Combined effects of climate change and LULC on sediment yield	Integrated simulation using both projected climate (A, B, and C) and projected LULC (2024–2064) across both SSP scenarios.

#### 2.9.2. Statistical Analysis of Land Use Change and Climate on SY and Hydrology

Generally, the procedures of statistical evaluation of impacts on land usage and climate change rely on predictions through modeling for the corresponding influence of SY on developing human activities and precipitation changes. Future scenario modeling will be based on the period from 2024 to 2064. The following equation was used for the quantitative computation of percent changes in SY and SR under different SSPs:

%change=final year value−starting year valuestarting year value∗100.

## 3. Results and Discussion

### 3.1. Climate Change

#### 3.1.1. Historical Climate Trends

For the interval between 1986 and 2014, the Baro River sub‐basin recorded a Mann–Kendall *Z*‐value of 0.13. Table 9 shows an increase in the rainfall volume, but the trend is not significant, indicating that the rainfall volume remained constant without shifting toward either wet or dry conditions. In addition, the low coefficient of variation (CV) of 6.75% further indicates consistency in the rainfall volume. This is in line with the results of Worku et al. [72], who reported that trends in rainfall volume in different agro‐ecological zones in Ethiopia often lack significant long‐term directionality. Both the maximum (26.64°C) and minimum (16.56°C) temperatures had substantially increasing trends, with *Z*‐scores of 2.91 and 2.95, respectively (*p* < 0.01). These results confirm warming and are in line with regional climate change in East Africa. These trends have been reported previously in the Jemma sub‐basin and the Blue Nile region, as reported by Worku et al. [72].

**Table 9:** Statistical analysis results of historical annual precipitation and temperature trends over the Baro River sub‐basin (1986–2014), significant at  ^∗^ *p* < 0.01.

	Average value	CV	*Z*‐value
*P*	1583.4 mm	6.75	0.13
Tmax	26.64°C	1.07	2.91 ^∗^
Tmin	16.56°C	1.67	2.95 ^∗^

**Figure 3:**
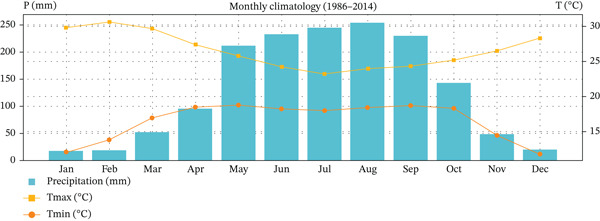
Multi‐year annual precipitation and temperature cycle during the historical period 1986–2014 over the Baro River sub‐basin.

The multi‐year monthly analysis of the Baro River sub‐basin, shown in Figure 3, for the historical period 1986–2014, shows a clear unimodal rainfall pattern and a stable temperature regime. The rainy season extends from May to October. Precipitation begins increasing from April and peaks in July and August, and the dry season occurs from December to February, during which the monthly precipitation falls below 21.7 mm. The strong concentration of precipitation during June, July, August, and September is attributed to the northward migration of the ITCZ. As noted by Mengistu et al. [36], the Baro–Akobo region is one of the wettest parts of Ethiopia, a vital “water tower” feeding into the White Nile. The mean monthly temperature varies less than precipitation but follows a definite seasonal decline. The maximum temperatures occur during the pre‐monsoon season in March and April, and minimum temperatures occur in August at the peak of the wet season, primarily due to dense cloud cover and elevated humidity levels. As illustrated in Figure 3, temperature and precipitation show a clear inverse relationship characteristic of the Ethiopian highlands. The decline in temperature during the peak rainy period (July–August) is attributed to the reduction in solar radiation reaching the Earth’s surface due to cloud cover.

#### 3.1.2. Projection of Precipitation and Temperature

##### 3.1.2.1. Precipitation Projection

**Figure 4:**
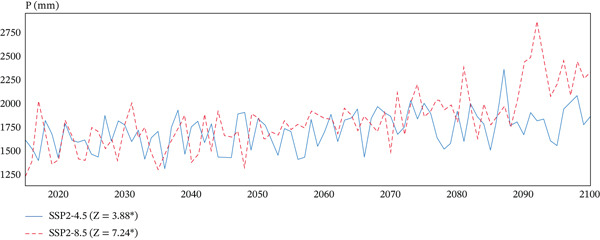
Projected annual precipitation (2015–2100) for the Baro River basin across two climate scenarios. Bracketed *Z*‐values indicate results from the Mann–Kendall trend test (significant at  ^∗^ *p* < 0.05).

Figure 4 illustrates projected annual precipitation for the study area from 2015 to 2100. These results represent a 12‐model ensemble under both medium‐emission (SSP2‐4.5) and high‐emission (SSP5‐8.5) scenarios. Both scenarios exhibit an increase in rainfall throughout the 21st century; however, the variation and magnitude differ considerably. For SSP2‐4.5, the annual rainfall maintains a steady rising trend with fluctuations from around 1500 mm in the initial phase to around 2000 mm by the end of the period. A Mann–Kendall trend investigation confirms a rising trend in the data, with a*Z*‐value of 3.88 (*p* < 0.05). SSP5‐8.5 shows a significantly steeper rise with greater variability, mainly after 2080, such that the annual rainfall reaches over 2750 mm in certain years.

It shows a highly significant rising trend in the data, with a *Z*‐value of 7.24 (*p* < 0.05), indicating an increased hydrological response due to high‐emission forcing. Results show that the basin would undergo a rise in rainfall in the future according to the two scenarios but at significantly different levels of intensity and variation, where SSP5‐8.5 would be linked with higher levels of intensity and variation, with the potential to enhance flood risk, alter water availability, and affect water‐dependent functions.

**Figure 5:**
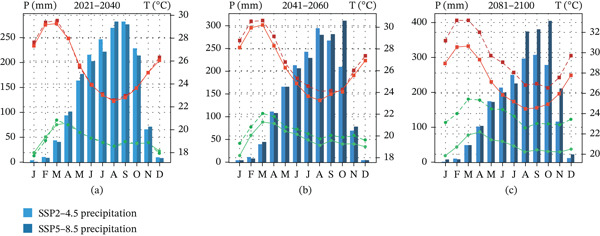
Projected precipitation trends across three future time periods, the near‐term (2021–2040), mid‐term (2041–2060), and late‐term (2081–2100) periods, under SSP2‐4.5 and SSP5‐8.5 scenarios in the Baro River sub‐basin.

Figure 5 shows a clear seasonal pattern of precipitation that is common for all periods of the study. The highest precipitation is expected during the months of August–October. It is noteworthy that both scenarios demonstrate almost equal precipitation patterns during the near‐term period (2021–2040); however, a notable difference is seen in late‐century projections (2081–2100). For instance, according to the SSP5‐8.5 scenario, the maximum monthly precipitation might reach almost 400 mm, while in the mid‐century period, only 275 mm of monthly precipitation is forecasted. As can be seen from Figure 5, there is a continuous increase in precipitation throughout the entire 21st century. Within the next two decades (2021–2040), the annual precipitation is expected to grow by 5%–8% and 10%–12% according to SSP2‐4.5 and SSP5‐8.5, respectively. The mid‐term period witnesses even greater precipitation increments (12%–15% and 20%–25% correspondingly). The highest precipitation changes are observed during the late‐term period (2081–2100). Annual precipitation is likely to grow by 20% under SSP2‐4.5 and by 35%–40% under SSP5‐8.5.

The projected average annual and seasonal precipitation under the SSP2‐4.5 and SSP5‐8.5 scenarios for the period 2021–2100 is compared against the historical baseline (1986–2014), as presented in Table 10. The MME indicates an overall increase in total annual precipitation across both scenarios. From a historical baseline of 1583.43 mm, annual rainfall is projected to rise to 1715.40 mm (+8.3%) under SSP2‐4.5 and 1821.91 mm (+15.1%) under SSP5‐8.5. The MME projections indicate a general upward trend in annual precipitation, though the distribution across the three seasons, Bega, Belg, and Kiremt, varies significantly. Kiremt (main rainy season) is the primary contributor to annual totals, showing a robust increase from 821.45 to 980.27 mm under the SSP5‐8.5 scenario. Bega (dry season) shows the highest relative growth, with projections suggesting a significant wetting trend, increasing by over 110 mm in the SSP5‐8.5 scenario compared to the historical period. Belg (short rainy season), conversely, is the only period projected to experience a decline. Precipitation is expected to drop from 608.33 mm to approximately 570–575 mm, representing a decrease of roughly 5.4%–6.3%. The projected increase in total annual precipitation is consistent with the latest IPCC Sixth Assessment Report (AR6) findings for East Africa, which suggest that a warming atmosphere increases moisture convergence and intensifies the hydrological cycle [73]. The substantial increase in Kiremt rainfall suggests that projected climate change may lead to further frequent and intense precipitation events [74].

**Table 10:** Average seasonal precipitation classification under different scenarios during 2021–2100 and the historical period 1986–2014 over the Baro River basin.

Season	Historical (mm)	MME	
		SSP2‐4.5	SSP5‐8.5
Bega	153.65	231.8	266.30
Belg	608.33	570	575.33
Kiremt	821.45	913.6	980.27
Annual	1583.43	1715.40	1821.91

##### 3.1.2.2. Temperature Projection

**Figure 6:**
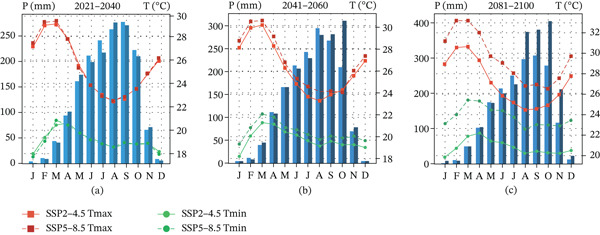
Intra‐annual distribution of future temperature (Tmax and Tmin) in the annual cycle under different climate scenarios and periods.

From the observations in Figure 6, there is a clear trend toward increased temperature for both Tmax and Tmin in all scenarios and time periods. For the near‐term period, Tmax is projected to increase by 1.2°C (SSP2‐4.5) and 1.8°C (SSP5‐8.5). At the same time, Tmin is projected to increase by 1.0°C (SSP2‐4.5) and 1.5°C (SSP5‐8.5). In the mid‐century period (2041–2060), the increase in warming becomes more pronounced, with an increase of 2.1°C (SSP2‐4.5) and 2.9°C (SSP5‐8.5) for Tmin. An increase in Tmin is also projected at 2.0°C and 2.7°C. In the late‐century period (2081–2100), the largest divergence is seen. While the projected Tmax and Tmin are expected to increase by 3.0°C each under the low‐emission SSP2‐4.5 scenario, Tmax is expected to rise by far more than 5.0°C and Tmin by nearly 5.0°C in the SSP5‐8.5 scenario.

The projections from 2021 to 2100 are shown in Table 11. One can clearly see that a significant warming trend exists across the study area for both SSP2‐4.5 and SSP5‐8.5 scenarios. The *p* value of < 0.01 confirms that a significant change exists. In the medium‐emission scenario, SSP2‐4.5, the average maximum temperature will rise to 26.31°C with a steady increase rate of 0.271°C per decade, and the minimum temperature will experience an increase at a rate of 0.293°C per decade. In the high‐emission scenario, SSP5‐8.5, the rate of change will rise sharply. The maximum temperature will rise to an average of 27.3°C, while the minimum temperature will rise to 21.12°C. It is important to note that the rate of change will be more than double that of the SSP2‐4.5 scenario. In SSP5‐8.5, the rate of change will be 0.745°C per decade, with Tmin having the highest rate of change. These results are in line with the latest AR6 findings from the IPCC, which indicate that the highlands and river basins of East Africa are likely to experience more frequent and intense heat events [73]. The significant *Z*‐test values obtained for the Baro River serve to reinforce the latest warnings from Molla and Melka [74], which indicate that the future rise in thermal stress and the decline in the Belg rains will pose a complex challenge to water resource management.

**Table 11:** Future projection of annual average temperature (2021–2100), change rate, and statistical trend analysis ( ^∗^ *p* < 0.01).

Temperature (°C)			Average values	Change rate (°C/decade)	*Z*‐value
MME	SSP2‐4.5	Tmax	26.31	0.271	9.44 ^∗^
		Tmin	19.90	0.293	10.11 ^∗^
	SSP5‐8.5	Tmax	27.3	0.609	11.56 ^∗^
		Tmin	21.12	0.745	11.95 ^∗^

### 3.2. Projected LULC Change

#### 3.2.1. Accuracy Assessment and Model Validation

##### 3.2.1.1. Accuracy Assessment

To conclude the reliability of the classification of the LULC and the predictive capacity of the model, the accuracy and validity of the findings from the study were evaluated thoroughly. Accuracy verification for the classified LULC was done for each period of the study: 2004, 2014, and 2024. Through the application of stratified random sampling, the accuracy of the classified LULC was found to be very high. The overall classification accuracy yielded results of 91.46%, 93.26%, and 94.46% for the years 2004, 2014, and 2024, respectively. High kappa coefficient agreement in the classifications was established, and the kappa coefficients were obtained as 0.90, 0.92, and 0.93, respectively. The accuracy of the classifications was extremely high (≥ 80%) and, therefore, highly acceptable [75, 76].

##### 3.2.1.2. CA‐MC Model Validation

The CA‐MC model was validated by associating the simulated LULC results for 2024 against the actual 2024 LULC classified map. The validation process focused on both the quantity of pixels in each class and their specific spatial locations. The model demonstrated a reasonable degree of agreement between the actual and predicted maps, confirming its reliability for future LULC change forecasting. The model’s performance was measured using various KIA (kappa index of agreement) statistics (Table 12).

**Table 12:** Kappa statistics for the CA‐MC model validation.

Statistics	Value (%)
*K*_standard_	87.63
*K*_location_	91.12
*K*_no_	88.42
*K*_locationStrata_	81.42

#### 3.2.2. Spatiotemporal LULC Dynamics

The analysis indicated that the landscape will predictably experience a significant transition from natural ecosystems to human‐modified environments between 2024 and 2064.

**Figure 7:**
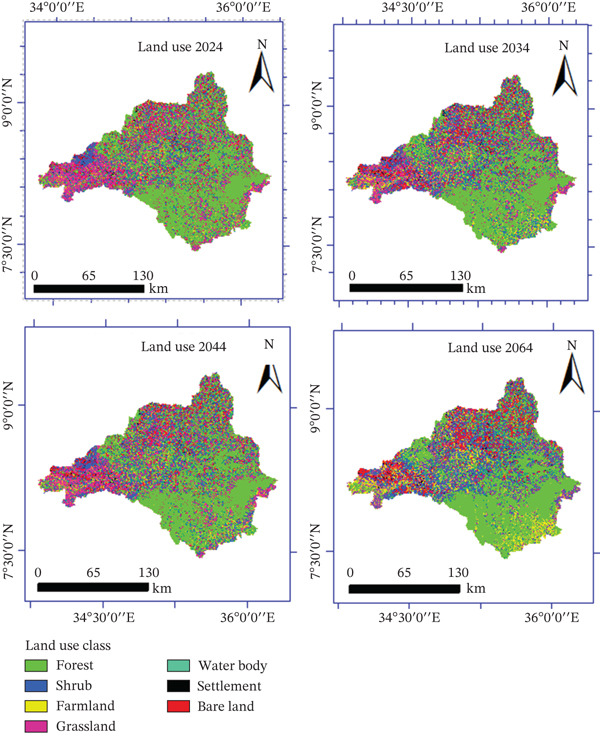
LULC maps of 2024, 2034, 2044, and 2064.

Based on Figure 7 and Table 13, forest lands are the main land use class; forest land is expected to decrease from 51.81% in 2024 to 39.00% in 2064. This shows a total reduction of 12.81% and indicates high rates of deforestation or land conversion and increasing anthropogenic pressure. Both land use classes, such as shrubland and grassland, show a steady downward trend. Grassland decreases from 8.77% to 5.84%, while shrubland falls from 10.28% to 7.03%. This trend indicates a high rate of LULC conversion. The decrease in forest land, grassland, and shrubland typically leads to the loss of and disruption to local watershed hydrology.

**Table 13:** Statistics for land use type changes.

Land use class	Land use 2024 (%)	Land use 2034 (%)	Land use 2044 (%)	Land use 2064 (%)
Forest	51.81	47.07	45.12	39.00
Shrub	10.28	9.18	8.00	7.03
Grassland	8.77	7.84	6.95	5.84
Farmland	24.42	28.73	30.06	35.49
Water body	0.51	0.47	0.43	0.39
Settlement	0.79	1.85	2.23	3.68
Bare land	3.42	4.86	7.22	8.57

Agricultural lands were the fastest‐growing land use class, expected to increase from 24.42% in 2024 to 35.49% in 2064. In 2064, agricultural land will occupy more than a third of the total land area, likely to meet the rapidly increasing expansion of human activity. This loss of vegetation land is directly offset by the increase in agricultural land and urban/settlement areas [77, 78]. From a baseline of 0.79%, urban or residential areas are expected to increase to nearly 3.68%. This indicates rapid urbanization and population growth [79]. Bare lands are expected to increase from 3.42% to 8.57%; this is due to deforestation and unsustainable farming practices leading to soil erosion or land degradation, ecological instability, and loss of soil fertility. Bare land refers to areas with no significant vegetation cover, often resulting from deforestation, overgrazing, or land abandonment [80]. Agricultural land will almost compete with forest land as the principal land use class. This suggests that the principal cause of deforestation in this area is likely agricultural expansion to meet the rapid expansion of human activity. The expansion of agricultural land and settlements at the expense of forests and grasslands suggests a high risk of ecological instability.

**Figure 8:**
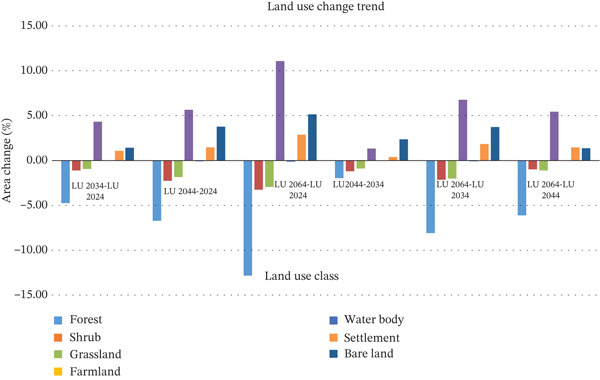
Gain and loss trends in land use change.

Figure 8 indicates that the farmland land use class exhibits the most considerable positive change, with a sharp increase of more than 10% projected for the LU 2064‐LU 2024 period. This significant increase likely occurs at the expense of other land types and is a common trend often driven by growing population and food demand. Vegetation cover, for instance, forest land, shrubland, and grassland areas, is steadily projected to be reduced across most timeframes. These declines, especially the significant negative percentages for forests and shrublands (e.g., in the LU 2064‐LU 2024 period), indicate a potential loss of natural habitats and biodiversity, which can have significant environmental consequences. The result is visible: forests, shrublands, and grasslands are under pressure and are being changed principally into agricultural land and, to a lesser extent, into settlements.

Based on Table 14, the results show that the first 10 years (2024–2034) see the most volatile changes. Settlement growth is explosive at 8.88% per year, suggesting a period of massive infrastructure investment or rapid urban migration. Similarly, agricultural land expansion is also at its peak (+1.64% annually), which directly correlates with the sharpest decline in forest (−0.96%) and grassland (−1.12%). From 2034 to 2044, forest land loss slows down slightly during this decade, whereas bare land expansion accelerates to its highest rate (4.04% per year). This is a red flag for land degradation. It suggests that the land being cleared is not all being successfully converted to productive farmland; instead, significant portions are becoming degraded or abandoned. In the final 20 years from 2044 to 2064, the rates of change generally slow down. This could be because there is simply less forest land and grassland left to convert or because the region reaches a point where most convertible land is already occupied by farms or buildings. While forest land receives the most attention, grassland and shrubland are actually disappearing at a faster average annual rate (−1.01% and −0.94%) than forest (−0.71%). These buffer ecosystems are often the first to be cleared for grazing or small‐scale farming, yet they are vital for local soil stability.

**Table 14:** Annual rate of change of land use in the study area.

Land use class	2024–2034 (10 years)	2034–2044 (10 years)	2044–2064 (20 years)	40‐year average
Forest	−0.96%	−0.42%	−0.73%	−0.71%
Shrub	−1.13%	−1.37%	−0.65%	−0.94%
Grassland	−1.12%	−1.19%	−0.87%	−1.01%
Farmland	1.64%	0.45%	0.84%	0.94%
Water body	−0.81%	−0.89%	−0.49%	−0.67%
Settlement	8.88%	1.88%	2.53%	3.92%
Bare land	3.58%	4.04%	0.86%	2.32%

### 3.3. Implication of Land Use and Climate Dynamics on SY

#### 3.3.1. Sensitivity Analysis, Calibration, and Validation

##### 3.3.1.1. Sensitivity Analysis

Table 15 ranks the 10 most sensitive parameters for simulating SY and SR in the Baro River sub‐basin, including their fitted values.

**Table 15:** Sensitivity analysis results including the minimum, maximum, and fitted values calibrated via Sequential Uncertainty Fitting Version 2 (SUFI‐2).

Rank	Parameter	Description	Fitted value	Min	Max
1	R_CN‐2	SCS (soil conservation service) runoff curve number for moisture condition‐II	0.2	−0.25	+0.25
2	V_ALPHA_BF	Base flow alpha factor (days)	0.674	0	1
3	R_CH_K2	Effective hydraulic conductivity of the main channel	101.72	0.05	500
4	R_ESCO	Soil evaporation compensation factor	0.0468	0.02	0.6
5	r__EPCO.hru	Plant uptake compensation factor	0.4	0	1
6	V_REVAPMN	Threshold depth of water in the shallow aquifer for “revap” to occur (mm)	438.19	0	500
7	R_SOL_K	Saturated hydraulic conductivity (mm/h)	1101.3	0	2000
8	r__CH_N2.rte	Manning’s roughness coefficient (main channel)	0.373	0	1
9	R_SOL_AWC	Soil available water capacity	0.472	0	1
10	R_RCHRG‐DP	Deep aquifer percolation fraction	0.216	0	1

##### 3.3.1.2. Calibration and Validation

**Figure 9:**
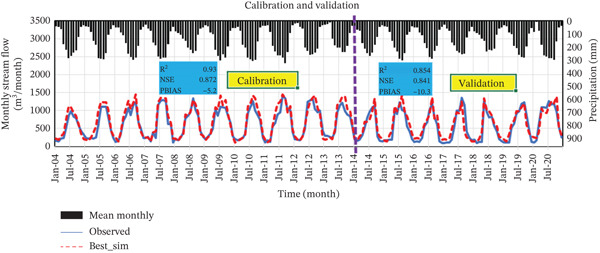
Observed versus simulated stream flow for the calibration and validation period.

**Figure 10:**
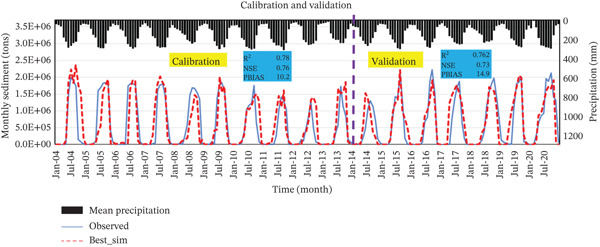
Observed versus simulated sediment for the calibration and validation period.

Sediment and stream flow calibration and validation were performed over a 17‐year period (2004–2020). The SWAT model simulation was calibrated using hydrological data from 2004 to 2013 and validated from 2014 to 2020. The model demonstrated a strong fit with observed data, and the performance *R* ^2^ and NSE coefficients are presented in Figures 9 and Figure 10. Overall, the simulated flow patterns align closely with observed trends.

#### 3.3.2. SY Responses to Individual and Integrated Effects of Climate and Land Use Change

##### 3.3.2.1. Impact of Climate Change on SY

The results of the simulations (see Table 16) demonstrate increasing SY in both climate scenarios compared to the base value of 49.23 t/ha. For the SSP4.5 scenario, the increase is expected to reach 56.78 t/ha in the short term (2015–2043), and it will reach its peak of 85.18 t/ha during the last years of the century (2072–2100). The SSP8.5 scenario shows the most radical growth, with the doubling of SY in the mid‐term period (2044–2071), where the amount will be 94.48 t/ha, and it will reach 107.26 t/ha in 2100. In comparison with the base climatic scenario, the high‐emission scenario (SSP8.5) will result in a 56% increase in sediment production within the same time period used in the land use planning process. Moving from SSP4.5 to SSP8.5 causes an increase in sediment production. Also, SSP4.5 will cause more sediment production than the base scenario. The notable response at the end of the century may reflect the enhanced effects of greenhouse gas forcing on rainfall intensities, storms, and runoff, leading to increased shear stress on the soil surface, thus increasing the detachment and transport capacity. Several scientific literature sources show that the relationship between soil erosion and rainfall erosivity is nonlinear, such that slight increases in rainfall intensities may result in disproportionate increases in SY [81, 82]. Although the results match the regional trends in East Africa, the steeper projected increases and higher baseline yield suggest greater vulnerability compared to other semi‐arid studies. These predictions correlate well with other predictions for other sub‐basins within the Nile River basin, where there will be notable increases in erosion and SY during the latter part of the century [83].

**Table 16:** Impact of SSP4.5 and SSP8.5 climate scenarios on projected sediment yield using 2024 land use data.

Land use	Climate scenario		Sediment yield (t/ha)
LULC 2024	Baseline	1986–2014	49.23
	SSP4.5	2015–2043	56.78
		2044–2071	62.79
		2072–2100	85.18
	SSP8.5	2015–2043	64.72

##### 3.3.2.2. Impact of Land Use Change on SY

Based on Table 17, which shows each single climate scenario, SY increases significantly as land use evolves from the 2024 baseline data to the year 2064. For example, under baseline climate conditions (1986–2014), SY shows a constant increasing pattern from 49.23 to 91.35 t/ha in the years 2024 and 2064, respectively. It is worth noting that despite differences in climatic scenarios, there seems to be a consistent increase in SY caused by alterations in land use patterns such as deforestation and urbanization. For example, based on LULC change only, in 2064, the percentage increase in SY is 85.5%.

**Table 17:** Projected mean annual sediment yield variations across LULC and baseline climate change.

Baseline climate (1986–2014)	LULC (year)			
	2024	2034	2044	2064
Sediment yield (t/ha)	49.23	68.92	79.13	91.35

The increase in SY is mainly due to the change from vegetated landforms such as forests, grassland, and shrubs to man‐made areas such as cultivated areas and settlements. The cultivated area shows high soil loss relative to other LULC categories due to disturbance and the absence of permanent cover. The consistent increase in SY due to land use change within each climate scenario indicates that expansion of cropland, reduction of forest and shrubland, and intensified cultivation on fragile soils are key drivers, in line with numerous Ethiopian and East African studies [33, 84, 85]. The conversion from forests or shrubs to agricultural lands increases the CN value as well as the *C* value, resulting in the percentage increase in SY from 49.23 to 91.35 t/ha in the year 2064.

##### 3.3.2.3. Combined Effects of Climate and LULC Change on SY

**Figure 11:**
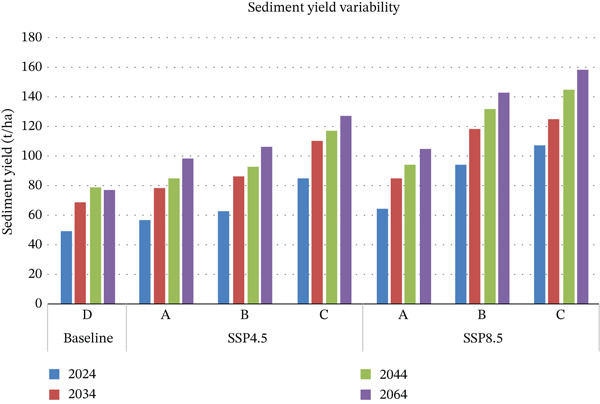
Comparison of projected sediment yield variability (t/ha) across multiple land use and SSP projections in the Baro River basin.

The overall results in Table 18 and Figure 11 indicate that the effects of land use and climate change compound SY. From the results, it is clear that the minimum amount of SY is observed when the land use and climate remain at baseline conditions, while the maximum SY of 142.52 t/ha is observed in the year 2064 when the land use and climate scenario correspond to SSP8.5 (2044–2071). It is evident that the combined effect of climate change makes the effects of land use changes much worse. For instance, although land use changes without climate change effects lead to an increase in SY from 49.23 to 91.35 t/ha by 2064, the inclusion of SSP8.5 effects turns that same land use scenario into one yielding 142.52 t/ha.

**Table 18:** Summary of projected mean annual sediment yield (t/ha) within the study area, categorized by land use and climate scenarios.

Climate scenario		Land use			
		2024	2034	2044	2064
Baseline	1986–2014	49.23	68.92	79.16	91.35
SSP4.5	2015–2043	56.78	78.31	84.49	98.59
	2044–2071	62.79	86.24	92.68	107.29
	2072–2100	85.18	110.33	117.33	126.33
SSP8.5	2015–2043	64.72	84.88	93.23	104.74
	2044–2071	94.48	118.15	131.64	142.52
	2072–2100	107.26	125.2	144.79	157.68

In turn, SSP4.5 produces more sediment than the base scenario in all timeframes, starting from 56.78 t/ha (2015–2043) and ending with 85.18 t/ha (2072–2100) in 2024 and from 98.59 to 126.33 t/ha in 2064. Thus, the increase in sediment production can be viewed as an acceleration due to increasing temperature and rain intensity. However, with high emissions (SSP8.5), there is an even more notable change in SY. During 2015–2043, SY is above baseline and SSP4.5 (64.72–104.74 t/ha). From 2044 to 2071, there are sharp increases to 94.48–142.52 t/ha, followed by further increases to 107.26–157.68 t/ha from 2072 to 2100.

The interaction between land use changes and climate change produces a synergistic effect, whereby the combined effect is greater than the individual effects of each factor alone. As described by Asselman et al. [86], changes in land use determine the availability of sediment from the process of soil erosion, while climate change, particularly the increase in rainfall intensity, determines the energy needed to move that sediment. In this research, the shift to the 2064 land use scenario will involve a decrease in surface roughness and infiltration capacity. When such degraded land is subjected to the more intense hydrological cycles projected under SSP8.5, it leads to an exponential increase in the rate of soil erosion.

The consistent increase in SY between land use change within each climate scenario indicates that expansion of cropland, reduction of forest and shrubland, and intensified cultivation on fragile soils are key drivers, in line with numerous Ethiopian and East African studies [84, 85]. The study area is known for rapid agricultural development and deforestation at the forest–agriculture boundary; similar boundary dynamics have elsewhere been associated with strong increases in hillslope erosion and channel SL. The results thus reinforce the conclusion that unmanaged land use change can amplify the erosive impact of climate change.

#### 3.3.3. SR Responses to Separate and Combined Impacts of Climate and Land Cover Dynamics

##### 3.3.3.1. Impact of Climate Change on SR

In hydrology modeling, Table 19 shows it is clearly visible that there is a trend of continuous growth in the annual SR in all future time periods and scenarios as compared to the base time period. SR is expected to rise from 639.07 mm in the historical period (1986–2014) of the constant LULC 2024 baseline scenario to a peak toward the end of the century (2072–2100). Under the medium‐emission scenario (SSP4.5), SR is seen increasing to 670.33 mm (+4.89%) in the near‐term period (2015–2043) and 694.81 mm (+8.72%) in the mid‐term period (2044–2071), and it reaches a peak value of 719.17 mm (+12.53%) by the end of the century (2072–2100). Likewise, the SR under the high‐emission scenario (SSP8.5) intensifies at a higher rate. SR under SSP8.5 increases to 691.76 mm (+8.24%) in the near‐term period and 708.05 mm (+10.79%) in the mid‐term period, and it peaks at 741.78 mm (+16.07%) in the later part of the century using LULC 2024. A comparison of the scenarios shows that the medium‐emission SSP4.5 scenario is always lower than the high‐emission SSP8.5 scenario for any corresponding period. For instance, the projection of SR for the near‐term period (2015–2043) under the SSP8.5 scenario is 691.76 mm, exceeding the SSP4.5 projection of 670.33 mm by 21.43 mm.

**Table 19:** Surface runoff dynamics under changing climate scenarios.

Land use	Climate scenario		Surface runoff (mm)
LULC 2024	Baseline	1986–2014	639.07
	SSP4.5	2015–2043	670.33
		2044–2071	694.81
		2072–2100	719.17
	SSP8.5	2015–2043	691.76

The interaction between a static landscape (LULC 2024) and deteriorating climatic conditions reveals that, irrespective of any additional deforestation activities, climate change could alone lead to an additional 16.07% of catchment degradation. Should changes in future land use, such as the conversion of forested areas into farmland, be included in this analysis, real‐world estimates of SY are likely to be much greater.

The results indicate that with regard to changes expected in climatic conditions using CMIP6 model simulations, the climate change projections for southwestern Ethiopia reflect an increase in annual average rainfall along with extremes in daily rainfall amount under SSP4.5 and SSP8.5 scenarios [87, 88]. Increased rainfall leads to an increase in rainfall erosivity, which breaks up soil components rapidly. The detachment of soil components increases the carrying capacity of water, which carries more soil into the Baro River. The results confirm that climate change will significantly worsen soil erosion and sediment transport within the Baro River sub‐basin. The positive trend over the three temporal blocks matches similar hydro‐sedimentological assessments conducted in East African highland catchments. For instance, studies by Ebissa et al. [89] and Muleta [90] emphasize that the southwestern Ethiopian plateau is highly vulnerable to extreme climate shifts.

##### 3.3.3.2. Impact of LULC Change on SR

The results of the runoff that would be produced on the surface of the Baro River sub‐basin under baseline climatic conditions and various LULC changes are presented in Table 20. From the table, the runoff increases steadily under baseline climate (1986–2014) from 639.07 mm in 2024 to 788.34 mm in 2064. The findings reveal that the runoff is anticipated to increase under baseline climate conditions. Within a span of 40 years, changes in LULC have caused an increase in runoff of 149.27 mm (+23.36%) by 2064 from the baseline climate era. The findings are consistent with the fact that a 16.5% expansion in agricultural area increased SR by 32 mm [91]. This gradual increase toward a runoff volume of 788.34 mm is a major threat not only environmentally but also socioeconomically.

**Table 20:** Surface runoff dynamics under changing climate scenarios.

Baseline climate (1986–2014)	LULC (year)			
	2024	2034	2044	2064
Surface runoff (mm)	639.07	742.98	771.41	788.34

This gradual increase in SR in the Baro River sub‐basin signifies a worsening capability for retaining water in the basin. In southern and southwestern parts of Ethiopia, this trend has been observed to be associated with significant anthropogenic influences on the land [91, 92]. A higher surface water flow rate will increase the chances of floods downstream, degradation of the riverbanks, and soil erosion. Moreover, since more water will be lost through SR, there will be a decrease in the replenishment of the water table. This will put the dry season flows at risk of depletion.

##### 3.3.3.3. Combined Effects of Climate and LULC Change on SR

**Figure 12:**
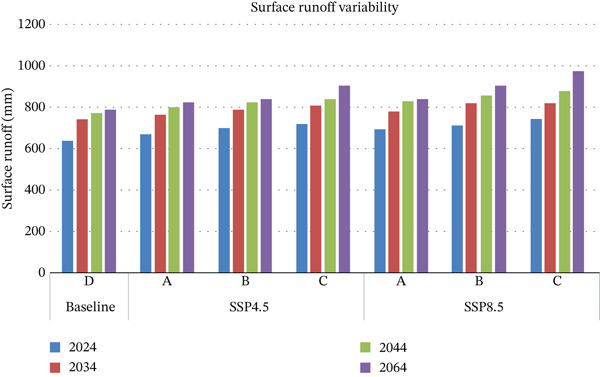
Comparison of projected surface runoff variability (mm) across multiple land use and SSP projections in the Baro River basin.

Table 21 and Figure 12 present the simulated impacts of climate and land use change on the average annual SR within the Baro River sub‐basin. Utilizing a calibrated SWAT hydrological model, these projections incorporate CMIP6 climate scenarios (SSP4.5 and SSP8.5) alongside land use map projections for 2034, 2044, and 2064, evaluated against a 2024 baseline.

**Table 21:** Projected mean annual surface runoff under changing climate and LULC.

Climate scenario		Land use			
		2024	2034	2044	2064
Baseline	1986–2014	639.07	742.98	771.41	788.34
SSP4.5	2015–2043	670.33	758.88	795.86	822.62
	2044–2071	694.81	792.79	820.53	839.1
	2072–2100	719.17	809.98	837.49	903.73
SSP8.5	2015–2043	691.76	779.31	826.75	842.4
	2044–2071	708.05	815.74	853.29	899.59
	2072–2100	741.78	818.39	875.39	966.58

The effect of both climate change and changes in LULC on the Baro River sub‐basin continues to elevate the SR (mm). Under the static climate baseline scenario, when the land use changes from 2024 to 2064, the runoff is projected to increase from 639.07 to 788.34 mm. SSP4.5 scenario trends show that the runoff is observed to increase with time. It reaches its highest level of 903.73 mm for climate periods of 2072–2100 with LULC conditions of 2064. SSP8.5 scenario trends show that the total runoff is highest under the extreme SSP8.5 scenario, increasing from its lowest value of 691.76 mm (for climate period 2015–2043 and LULC 2024) to its highest of 966.58 mm (for climate period 2072–2100 and LULC 2064). With the land use of 2064 held constant, the runoff increases from 788.34 mm under baseline climate conditions to 903.73 mm under SSP4.5 and 966.58 mm under SSP8.5. In the late‐century SSP8.5 scenario climate (2072–2100), changes in the land cover, from the 2024–2064 scenario period, result in a huge increase in runoff, from 741.78 to 966.58 mm.

SSP8.5 runoff results are always greater than SSP4.5 runoff results for all corresponding years of land use and climate scenarios. As a result of expanding impermeable/clear‐cut areas and increased rainfall, maximum runoff is achieved when the far‐future climate scenario coincides with the 2064 land use scenario.

The significant increase in SR indicates the very high vulnerability of the sub‐basin to multiple environmental threats. This synergistic effect witnessed in this case study conforms to the findings from hydrologic studies conducted in the Upper Blue Nile basin, showing that deforestation intensifies the effects of climate peak flows [93–95].

Peak runoff levels of 966.58 mm, as expected under SSP8.5, pose considerable dangers with regard to floods, enhanced soil erosion, and deposition within the reservoirs. Such a situation would be detrimental to local agricultural output and water resource management beyond the basin’s boundaries. As such, adopting ecosystem recovery practices, such as agroforestry and soil water conservation measures, becomes important [96].

#### 3.3.4. Comparative Analysis of Percentage Changes in SY and SR

Table 22 shows that SY increases by 57.12% and SR by 15.85%, indicating that even under baseline conditions (mainly land use/management change and internal climate variability), the basin becomes more erosive and generates more runoff. This suggests progressive vegetation loss, soil degradation, and expansion of cultivated or bare lands, which reduce infiltration capacity and soil cohesion [85, 97].

**Table 22:** Percentage change of SY and SR (2024–2064) over the Baro River basin.

Scenario	SY % change	SR % change
Baseline	57.12%	15.85%
SSP4.5 (2015–2043)	73.64%	22.72%
SSP4.5 (2044–2071)	70.87%	20.77%
SSP4.5 (2072–2100)	48.27%	25.65%
SSP8.5 (2015–2043)	61.84%	21.78%
SSP8.5 (2044–2071)	50.85%	27.05%
SSP8.5 (2072–2100)	47.01%	30.31%

Abbreviations: SR, surface runoff; SY, sediment yield.

Under medium‐emission climate change (SSP4.5), SY change ranges from 48.27% to 73.64%, and SR change ranges from 20.77% to 25.65%. The highest SY increase (73.64% for 2015–2043) indicates that early‐century warming and intensified rainfall events strongly enhance detachment and transport, especially where land use change has already reduced ground cover. The later period (2072–2100) shows a lower SY increase (48.27%) but the highest SR (25.65%), implying that rainfall intensity and soil crusting promote quick runoff generation, while possible sediment supply limitations or partial vegetation recovery may constrain further SY growth [64].

Under high‐emission conditions, SY increase is 61.84%–50.85%–47.01% while SR increases steadily from 21.78% to 30.31%. The monotonic rise in SR reflects stronger warming, more extreme storms, and higher rainfall intensity, which shorten concentration time and enhance overland flow [83]. However, the gradual reduction in SY % change (from 61.84% to 47.01%) suggests that, beyond a certain point, sediment production becomes supply‐limited: erodible topsoil is progressively removed, channel beds and banks adjust, and additional runoff does not translate proportionally into more sediment [98].

#### 3.3.5. Contribution of Land Use Classes to SY Under Combined Climate and LULC Scenarios

Table 23 presents the projected spatially averaged SY (SED, %) for each land use type (AGRL, agricultural land; RNGB, range brush/grass‐bush; BARR, bare land; FRST, forest; RNGE, range grass) under the baseline, SSP4.5, and SSP8.5 scenarios for the years 2024, 2034, 2044, and 2064. Over the entire period, AGRL always has the highest SED, with only small differences between the climate change scenarios. The SED of AGRL slightly declines from 31.88% (2024 baseline) to 25.14% (2044 baseline), then levels off at 25%–29% in 2064 but rises to 32.95% in 2064 under SSP8.5. BARR has high SED, reaching 28.80% in the 2034 baseline, then dropping to 18.35%–21.07% in 2064, particularly under SSP8.5, indicating some transformation of highly erodible but barren land. FRST has the lowest SED (~3%–4%) across all years and scenarios, thus emphasizing its strong erosion‐protective function. Rangeland types (RNGB and RNGE) have intermediate SED. RNGB remains at 21%–24% with low sensitivity to scenarios, whereas RNGE slightly increases over time in baseline and SSP8.5 scenarios, reaching ~24% in the 2064 baseline, indicating that degraded or overgrazed rangelands can become a significant sediment source.

**Table 23:** Relationship between land use type and sediment yield in the study site.

Land use type/class	Land use 2024			Land use 2034			Land use 2044			Land use 2064		
	SED (%)			SED (%)			SED (%)			SED (%)		
	Baseline	SSP4.5	SSP8.5	Baseline	SSP4.5	SSP8.5	Baseline	SSP4.5	SSP8.5	Baseline	SSP4.5	SSP8.5
AGRL	31.88	29.83	29.40	27.07	29.46	29.27	25.14	28.83	31.35	25.15	29.06	32.95
RNGB	20.84	23.36	23.56	20.62	23.46	24.32	23.32	23.50	23.70	22.94	23.24	22.58
BARR	23.53	22.49	21.26	28.80	21.07	19.71	25.81	22.13	18.86	24.23	21.07	18.35
FRST	3.55	3.17	2.86	3.45	3.15	3.11	3.31	3.56	2.95	3.50	3.72	3.35
RNGE	20.20	21.15	22.92	20.06	22.86	23.60	22.42	21.98	23.15	24.18	22.91	22.77

The prominent role of AGRL and BARR in SED reflects the general conclusion that the conversion of forest and rangeland to cropland and bare/urban land has a large effect on SY [99]. Results from the Mekong, Blue Nile, and Iranian watersheds indicate that the expansion of agricultural and developed land, in addition to increased rainfall amounts under RCP/SSP4.5‐8.5, results in 20%–200% increases in SY compared to past conditions [19, 28, 99]. The SED values from FRST support the conclusion that forest protection and afforestation can counteract climate change–induced erosion increases by increasing forest cover, litter, and root cohesion [100]. In some watersheds, the simulation scenarios that protect or increase forest area decrease SY despite increased rainfall erosivity and warming [19, 101]. The rangeland parameter in the table agrees with research indicating that the type and management of rangelands control their moderate sediment source or degraded bare land–like erosivity [102].

#### 3.3.6. Spatial Vulnerability of SY

**Figure 13:**
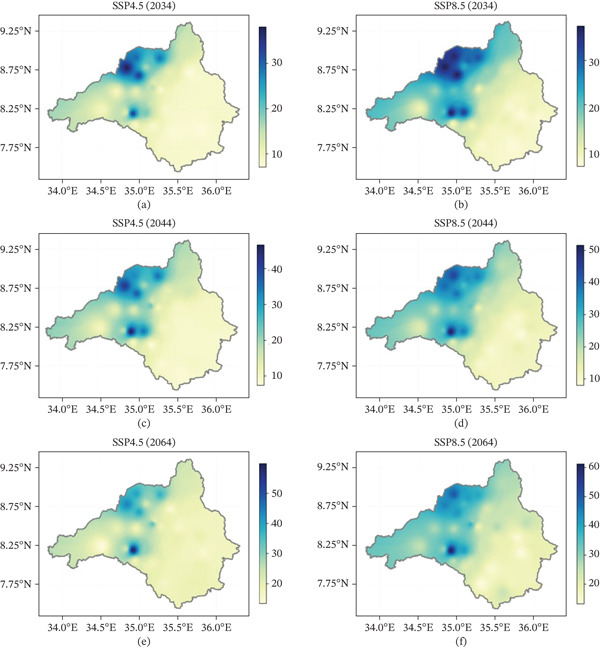
Impact of climate and land use change on sediment yield (t/ha/year).

Overall, based on Figure 13, the maximum SY is consistently intense in the north‐central and central regions, which appear as dark blue clusters. The eastern and southern sidelines of the Baro River sub‐basin show significantly lower SY, characterized by the light yellow/cream colors. There is a clear increase in SY over time. For instance, under SSP8.5, the maximum scale value increases from 40 t/ha/year in 2034 to 60 t/ha/year in 2064. At any given time step, SSP8.5 (right column) shows significantly higher peak SY relative to SSP4.5 (left column). By 2064, the hotspots in SSP8.5 cover a larger area and reach much higher magnitudes than in 2034.

The increase in SY from SSP4.5 to SSP8.5 suggests that increased temperatures, which typically lead to more intense rainfall events, are a primary driver of soil erosion in this study area. Higher precipitation intensity increases the erosivity of precipitation, detaching more soil particles and transporting them as sediment. These areas might be characterized by intensive agricultural expansion or deforestation, leaving the soil unprotected against the projected increase in rainfall intensity.

## Author Contributions

**Tewodros Getu Engida:** methodology, conceptualization, software, data curation, investigation, formal analysis, writing – original draft, visualization, writing – review and editing, validation. **Alemayehu Muluneh:** supervision, writing – review and editing, validation, resources, conceptualization, methodology. **Moltot Zewdie:** supervision, writing – review and editing, and conceptualization.

## Funding

No funding was received for this manuscript.

## Conflicts of Interest

The authors declare no conflicts of interest.

## Data Availability

The datasets used in this study were obtained from publicly available sources or are available upon request. The National Meteorological Institute (NMI) station observation data are available upon request from https://www.ethiomet.gov.et/. CHIRPS precipitation data are publicly available from the Climate Hazards Group InfraRed Precipitation with Station Data (CHIRPS) at https://www.chc.ucsb.edu/data/chirps. The projected climate data were obtained from publicly available CMIP6 datasets available at https://esgf‐metagrid.cloud.dkrz.de/search/cmip6‐dkrz/.
